# When Exophthalmos Reveals an Orbital Dermoid Cyst in a Child: A Case Report

**DOI:** 10.7759/cureus.102122

**Published:** 2026-01-22

**Authors:** Anas Douami, Daoud Bentaleb, Dalal Laoudiyi, Kamilia Chbani, Siham Salam

**Affiliations:** 1 Pediatric Radiology, Abderrahim Harouchi Mother-Child Hospital, Ibn Rochd University Hospital Center, Casablanca, MAR

**Keywords:** child, ct-scan, dermoid cyst, exophthalmos, mri

## Abstract

Orbital dermoid cysts are benign tumors most commonly found in young children. Although rare, their intraorbital location should not be overlooked. Imaging enables diagnosis and guides the surgical approach. We report the observation of an orbital dermoid cyst revealed by eye pain and unilateral exophthalmos of the left eye, in which imaging revealed an orbital cystic lesion whose radiological characteristics mainly suggest a dermoid cyst on computed tomography (CT) scan and magnetic resonance imaging (MRI). We report a case of an orbital dermoid cyst in a 10-year-old child.

## Introduction

Dermoid cysts are congenital benign tumors characterized by the presence of hair follicles, hair, sebaceous glands, keratinous material, or cholesterol crystals [1]. A dermoid cyst is a lesion with variable topography, either superficial (typically opposite the tail of the eyebrow) or intraorbital. Eighty percent are superolateral in topography [2,3]. Computed tomography (CT) and magnetic resonance imaging (MRI) allow for precise measurement of the location, size, and extent of dermoid cysts and help in planning the management of complex cases. We report a case of a left orbital dermoid cyst diagnosed on the basis of pathognomonic features observed on imaging, CT scan, and MRI.

## Case presentation

This concerns a 10-year-old patient with no particular medical history, who was seen in consultation for left eye pain, without any alteration of the general state or notion of fever. There was no history of trauma or ocular infection. Physical examination revealed exophthalmos in the left eye. There were no other associated signs, notably no neurological deficit or cervical adenopathy.

**Figure 1:**
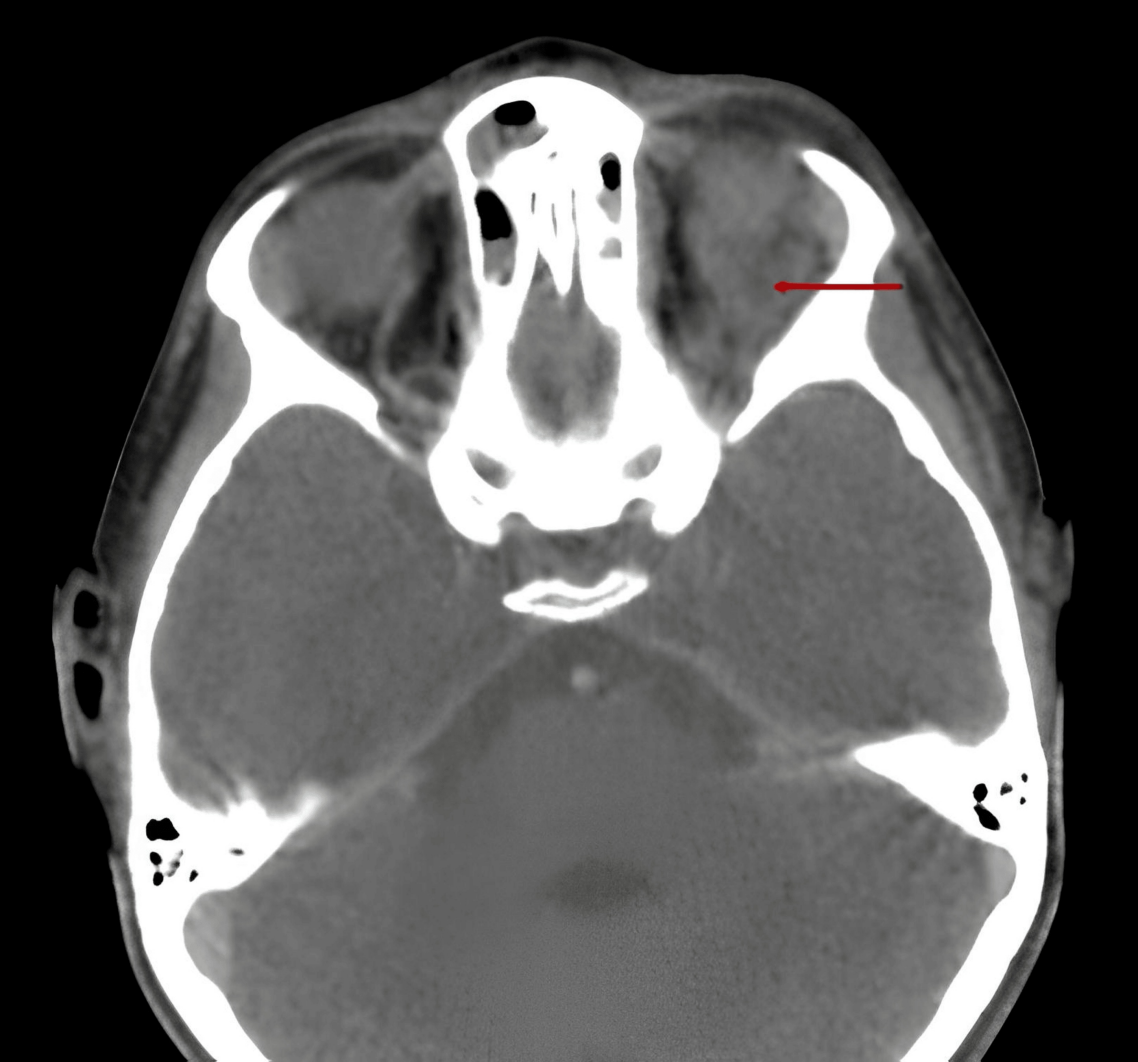
Axial scan with a parenchymal window in the left superolateral orbit, showing an oval-shaped, well-defined intraconal lesion with regular contours, fatty density, no enhancement after contrast injection, and no bone lysis (red arrow).

A facial CT scan was performed, showing, in the left superolateral orbit, an oval-shaped, well-defined lesion with regular contours and fatty density, not enhanced after injection of contrast agent and without bone lysis (Figure 1).

**Figure 2:**
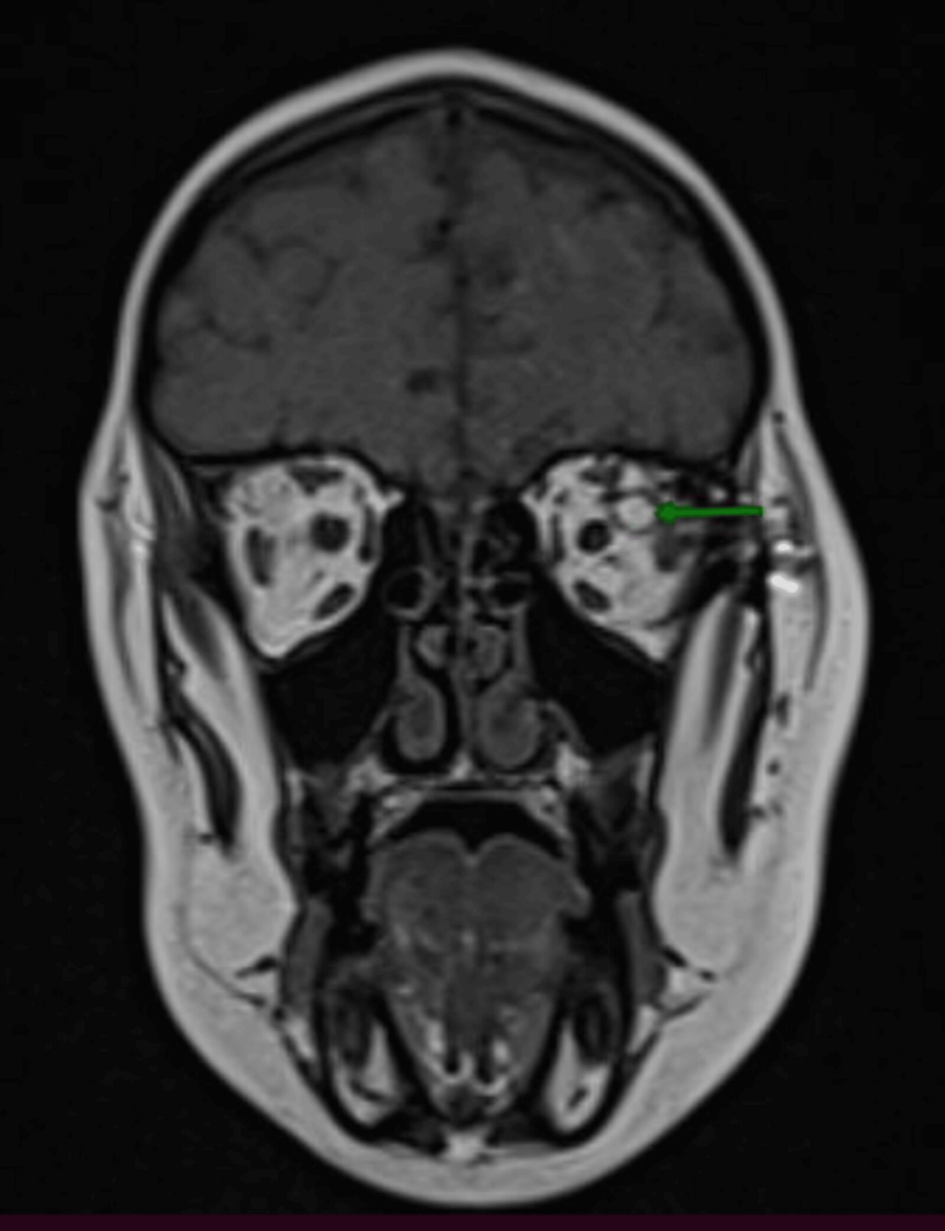
Coronal T1 MRI section revealing a well-circumscribed intraorbital conal mass located at the level of the left superolateral orbital wall, with hypersignal on T1 (green arrow). MRI, magnetic resonance imaging

**Figure 3:**
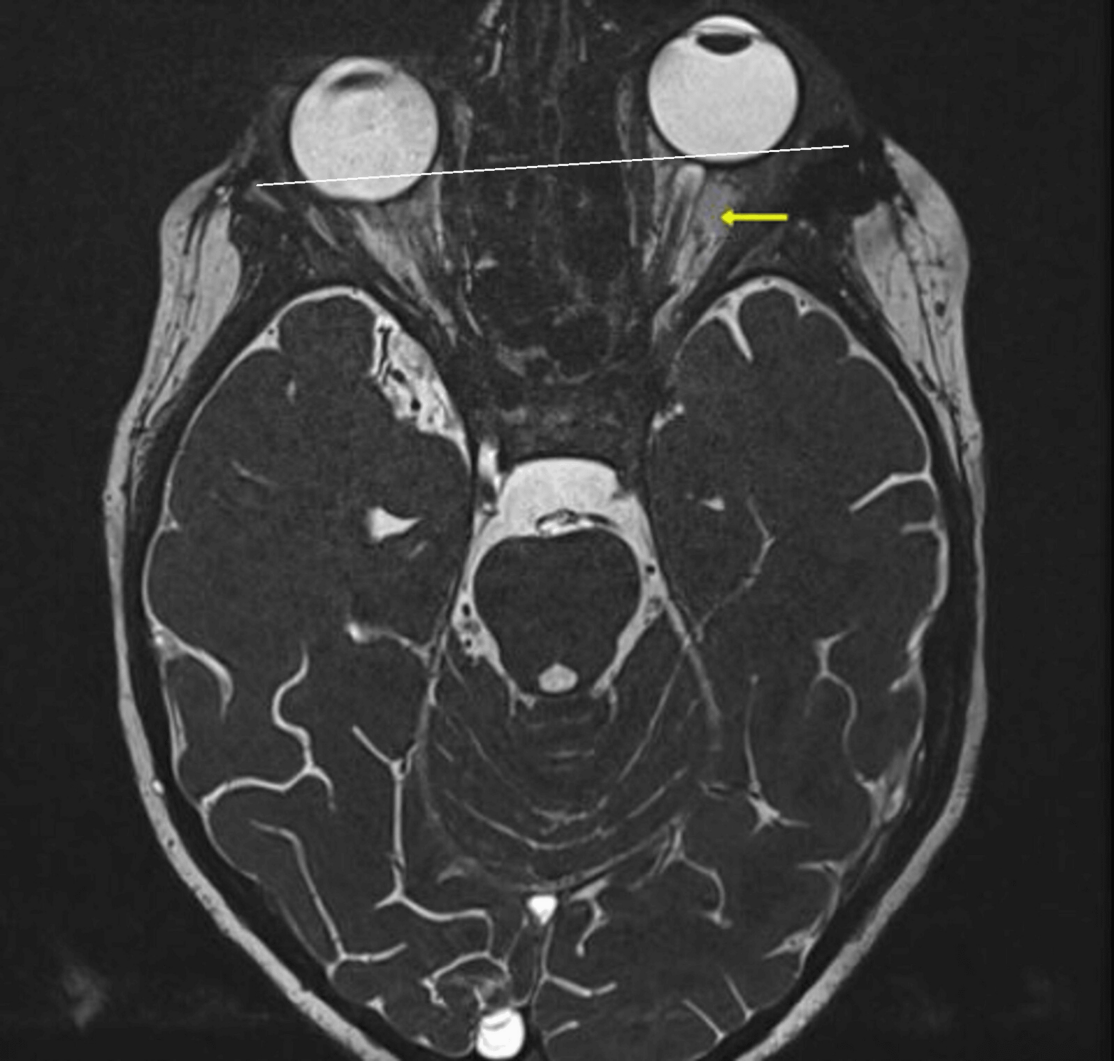
Axial T2 MRI section revealing a well-circumscribed intraorbital conal mass located at the level of the left superolateral orbital wall, with hypersignal on T2 (yellow arrow). It is responsible for grade I exophthalmos, as evidenced by the posterior third of the eyeball passing through the external bicanthal line (EBL). MRI, magnetic resonance imaging

**Figure 4:**
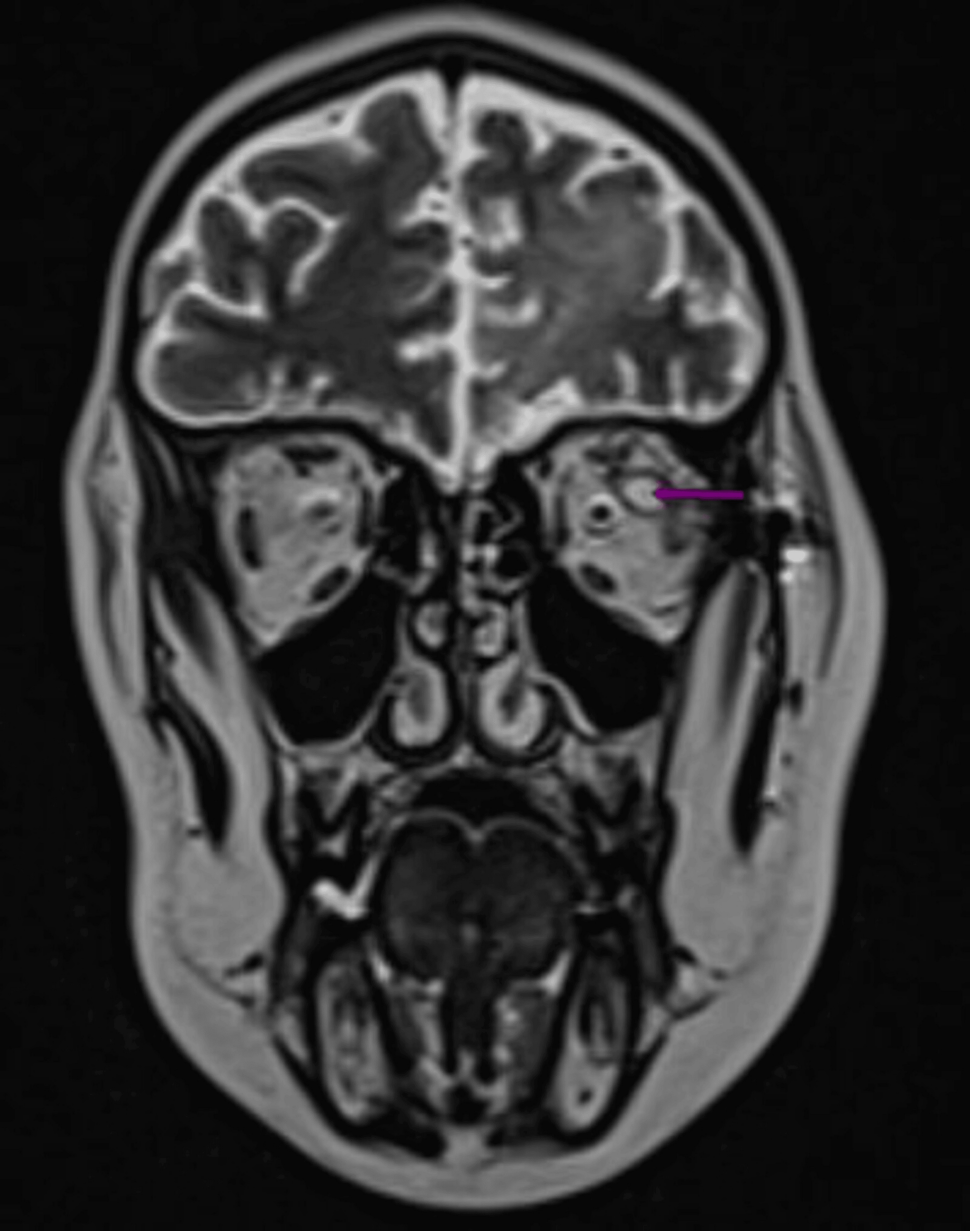
Coronal T2 MRI section revealing a well-circumscribed intraorbital conal mass located at the level of the left superolateral orbital wall, with hypersignal on T2 (purple arrow). MRI, magnetic resonance imaging

**Figure 5:**
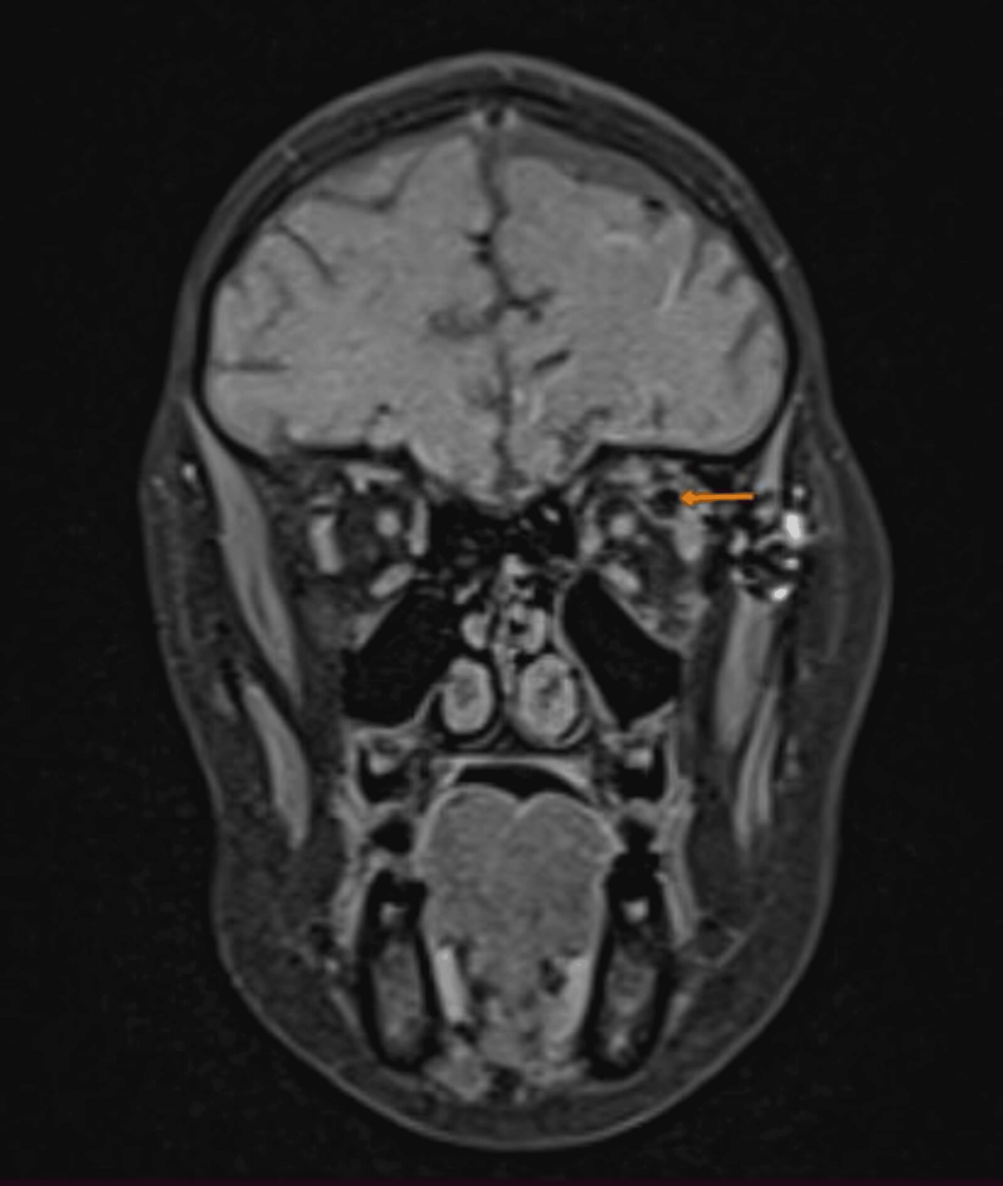
Coronal T1 fat-saturated MRI section showing signal suppression from an intraorbital mass in the left superolateral orbital wall, with hypersignal on T1 and T2 (orange arrow). MRI, magnetic resonance imaging

**Figure 6:**
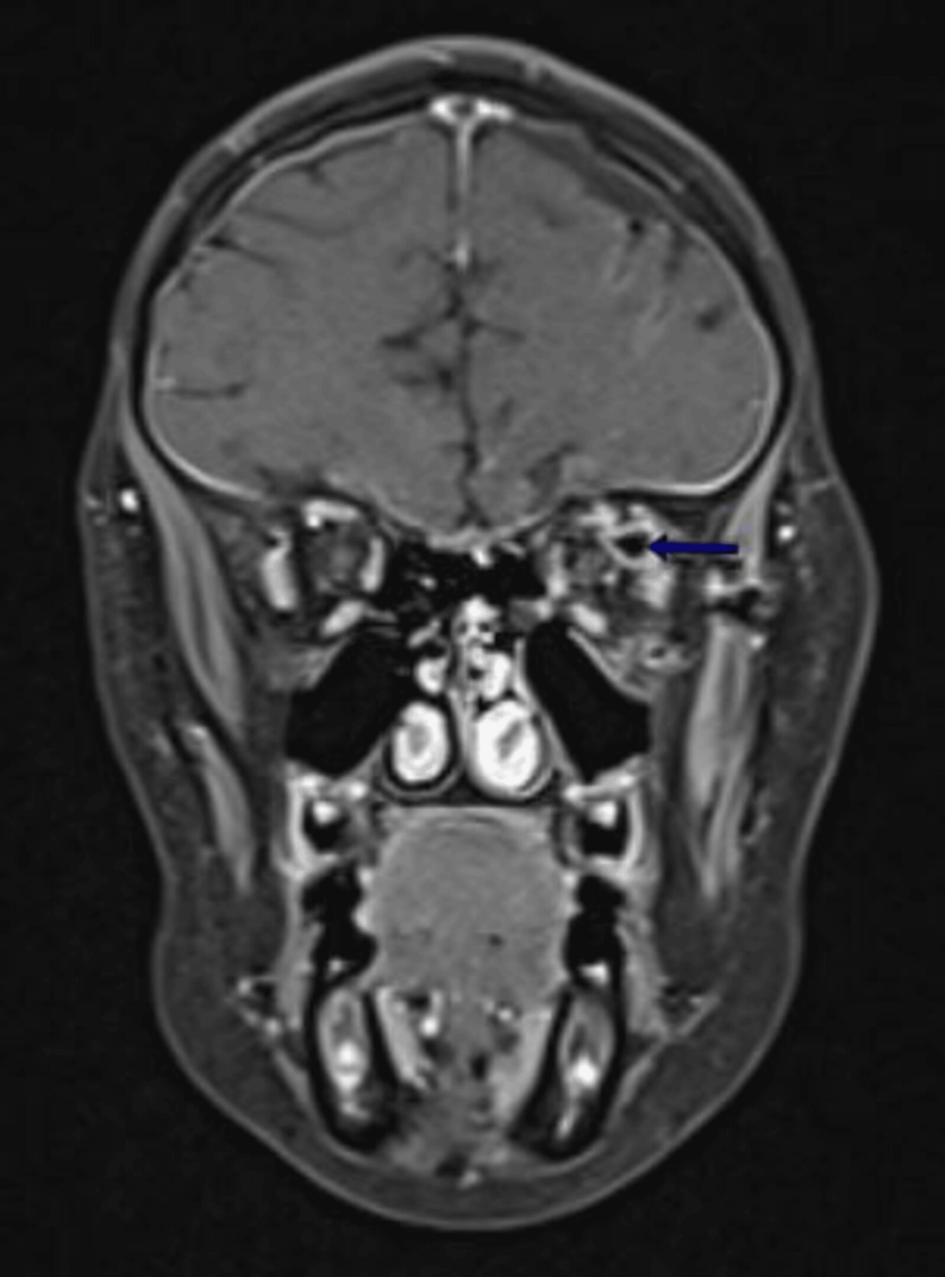
Coronal T1 fat-saturated (FAT SAT) MRI section after gadolinium injection, showing no enhancement of the mass (blue arrow). MRI, magnetic resonance imaging

**Figure 7:**
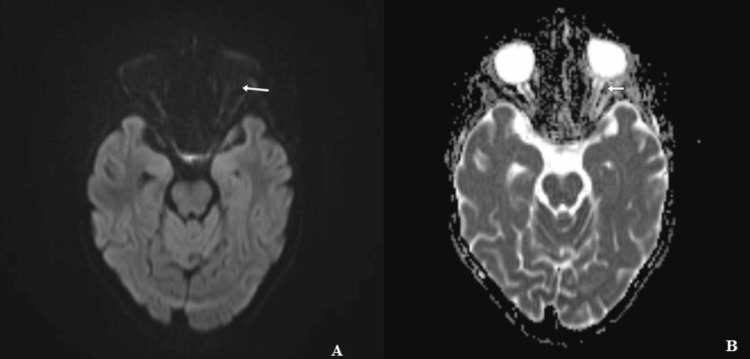
Diffusion sequence with its ADC cartography in the axial section, which does not demonstrate the restrictive character of the mass (white arrow). A) Diffusion at b1000; B) ADC cartography. ADC, apparent diffusion coefficient

Subsequent MRI confirmed a cystic mass in the left superolateral orbital wall, of fatty signal, hyperintense on T1 and T2, fading away on fat-saturation sequences, without diffusion restriction or enhancement with gadolinium (Figures 2-7).

It is responsible for grade I exophthalmos, as evidenced by the posterior third of the eyeball passing through the external bicanthal line (EBL). The preliminary diagnosis, based on CT and MRI findings, was in favor of an orbital dermoid cyst.

## Discussion

Dermoid cysts are benign congenital tumors [3]. They belong to the choriostromal group and result from the inclusion and isolation of a fragment of ectoderm (ectodermal nidus) in the subcutaneous tissue during neural tube closure, in contact with fetal sutures - in this case, the bone sutures of the orbit [4]. This fragment, incarcerated at the bone suture during the formation of the orbit, continues to grow autonomously and becomes clinically detectable during childhood, puberty, or later [5]. Dermoid cysts account for 14% of all orbital tumors and 20% of benign orbital tumors [6].

More than 80% of dermoid cysts are located in the head, most often in the eyebrow and orbital region, and more specifically in the superotemporal region near the frontozygomatic suture [1]. There are three types of dermoid cysts: sutural dermoid cysts, juxta-sutural dermoid cysts, and soft tissue dermoid cysts. They are generally classified as superficial dermoid cysts (simple and exophytic) and deep dermoid cysts (complicated and endophytic), according to their location in relation to the orbital septum [7,8]. The majority of dermoid cysts are superficial and are located more precisely at the tail of the eyebrow [5,9]. Diagnosis is therefore straightforward and is based on the presence of a firm, mobile swelling relative to the adjacent plane, adherent to bone structures. Only 0.5% of dermoid cysts are deep [9] and manifest late with exophthalmos when they increase in size during adolescence or adulthood [5,10].

Imaging is absolutely indicated when the clinical diagnosis is uncertain, or when a thorough assessment of the extent of the disease is necessary. The choice of technique depends on the clinical signs (location and mobility) [11].

When the mass is located at the tail of the eyebrow (typical case) and is very mobile, an ultrasound scan is nevertheless useful to confirm the diagnosis, measure the lesion, and study its relationship with the adjacent bone wall. The echostructure is variable, with the lesion often being heterogeneous, hyperechoic due to its fat content, sometimes less echogenic than the surrounding fat, but always non-vascularized on color Doppler. The contours should be perfectly clear and regular; a thick capsule may be present [11].

A CT scan is recommended as soon as the lesion is less mobile, more posterior, poorly defined on ultrasound, or medial, in order to study its relationship with bone and brain structures. The cyst is rounded, well-defined by a more or less thick capsule, sometimes calcified, with hypodense content of variable density (fluid or fatty), often heterogeneous, located opposite an orbital suture. Contrast injection is generally not necessary; if performed, there is no enhancement. The adjacent bone may be deformed, but its structure is generally normal [11].

MRI generally reveals dermoid cysts as well-defined, round or ovoid masses of varying sizes. The homogeneity of the mass influences its signal intensity; cysts may be iso- or hypointense on T1-weighted sequences, or may have a high signal intensity similar to that of fat, generally appearing hyperintense on T2-weighted sequences. Sometimes, the margin of the cyst may show calcifications, and the cyst wall may enhance in the event of inflammation. A decrease in diffusivity is expected, and a small fistulous tract may connect the cyst to the skin surface [12]. MRI is particularly valuable in delineating the extent of deep orbital cysts, which pose significant surgical challenges [13].

The differential diagnoses for dermoid cysts include orbital cellulitis, pseudotumor, epithelial inclusion cyst, hydatid cyst, and cold abscess. Imaging plays a key role in differentiating among these conditions [14].

The progression of dermoid cysts can be complex. The tumor may spread to the frontal sinus through the roof of the orbit, rupture spontaneously - causing a significant orbital inflammatory reaction and increased exophthalmos [12] - fistulate to the skin, or be complicated by oculomotor disorders or even compressive optic neuropathy [15]. The treatment of choice is complete surgical excision. This excision is sometimes difficult due to the frequency of adhesions to the periosteum [16].

## Conclusions

Dermoid cysts are the most common type of orbital cystic lesion reported in children. Clinical manifestations vary depending on their location and size. Imaging plays a crucial role in their diagnosis, helping to assess their extent. Raising public awareness of the importance of early and prompt treatment of these benign lesions is essential. Complete surgical excision remains the gold-standard treatment.
